# Safety of Inulin and Sinistrin: Combining Several Sources for Pharmacovigilance Purposes

**DOI:** 10.3389/fphar.2021.725417

**Published:** 2021-11-18

**Authors:** T-V. Bui, C. Prot-Bertoye, H. Ayari, S. Baron, J-P. Bertocchio, C. Bureau, P. Davis, A. Blanchard, P. Houillier, D. Prie, A. Lillo-Le Louet, M. Courbebaisse

**Affiliations:** ^1^ Assistance Publique-Hôpitaux de Paris, Centre Régional de Pharmacovigilance, Hôpital Européen Georges Pompidou, Paris, France; ^2^ Assistance Publique-Hôpitaux de Paris, Hôpital Européen Georges Pompidou, Service de Physiologie, Paris, France; ^3^ Centre de Référence des Maladies Rénales Héréditaires de l'Enfant et de l'Adulte (MARHEA), Paris, France; ^4^ Centre de Référence des Maladies Rares du Calcium et du Phosphate, Paris, France; ^5^ Centre de Recherche des Cordeliers, INSERM, Sorbonne Université, Université de Paris, Paris, France; ^6^ CNRS ERL 8228–Laboratoire de Physiologie Rénale et Tubulopathies, Paris, France; ^7^ Faculté de Médecine, Université de Paris, Paris, France; ^8^ Assistance Publique Hôpitaux des Hôpitaux de Paris, Hôpital Européen Georges-Pompidou, Centre d’investigation Clinique, Paris, France; ^9^ Institut National de la Santé et de la Recherche Médicale, Paris, France; ^10^ Service des Explorations Fonctionnelles, Hôpital Necker, APHP Centre-Université de Paris, Paris, France; ^11^ INEM Unité Inserm U1151, Paris, France

**Keywords:** inulin (PubChem CID: 16219508), safety, hypersensitivity, pharmacovigilance, sinistrin

## Abstract

**Introduction:** Inulin and its analog sinistrin are fructose polymers used in the food and pharmaceutical industries. In 2018, The French National Agency for the Safety of Medicines and Health Products (ANSM) decided to withdraw products containing sinistrin and inulin due to several reports of serious hypersensitivity reactions, including a fatal outcome.

**Objective:** To assess the safety of inulin and sinistrin use in France.

**Methods**: We searched multiple sources to identify adverse reactions (ARs) to inulin or sinistrin: first, classical pharmacovigilance databases including the French Pharmacovigilance (FPVD) and the WHO Database (VigiBase); second, data from a clinical trial, MultiGFR; third, data regarding current use in an hospital. All potential ARs to inulin or sinistrin were analyzed with a focus on hypersensitivity reactions and relationships to batches of sinistrin.

**Results:** From 1991 to 2018, 134 ARs to inulin or sinistrin were registered in the FPVD or VigiBase. Sixty-three cases (47%) were classified as serious, and 129 cases (96%) were hypersensitivity reactions. We found an association between a batch of sinistrin and the occurrence of hypersensitivity reactions. During the MultiGFR clinical trial, 7 patients (7/163 participants) had an Adverse reaction; of these, 4 were hypersensitivity reactions including one case of grade 4 anaphylactic shock. In the hospital, no ARs were observed. In the literature, ARs to inulin and sinistrin are very rarely reported and mostly benign.

**Conclusion:** Most ARs to inulin and sinistrin are hypersensitivity reactions that appear to be associated with sinistrin batches.

## Highlights

1) Inulin and its analog sinistrin are fructose polymers used in the food and pharmaceutical products.
2) Adverse reactions (ARs) to inulin or sinistrin have rarely been reported in the literature.
3) Most ARs to inulin and sinistrin are hypersensitivity reactions.
4) An association between some batches of sinistrin and the occurrence of these hypersensitivity reactions was found in our analysis.

## Introduction

Inulin and its analog sinistrin (also called polyfructosan) are fructose polymers produced by at least 36,000 plant species. They are used as energy reserves by plants and participate in the regulation of osmotic pressure and resistance to cold and drought (Cornelis, 2013; Shoaib et al., 2016)*.* Discovered in the early 1800s by the German scientist Valentine Rose, inulin has multiple applications in food and pharmaceutical industry. Inulin is used in processed foods as a prebiotic, as a fat or sugar replacement, and as a texture modifier (Shoaib et al., 2016). The uses of inulin in the pharmaceutical area are very diverse: It is a protein stabilizer and has been used in drug delivery formulations and as a stabilizer or adjuvant in vaccine formulations (Mensink et al., 2015a). Inulin and sinistrin are both used in diagnosis of kidney disorders. Renal clearances of sinistrin and inulin are considered as the gold standard for glomerular filtration rate (GFR) measurement. Neither are metabolized. They are freely filtered by the glomerulus and are neither secreted nor reabsorbed by the renal tubule. An advantage of sinistrin over inulin is its higher solubility in water, which results in easier handling and preparation for injection (Oettl et al., 2004; Sandilands et al., 2013).

Inulin is present in commonly consumed plants and is added to food products, and adverse effects of inulin or sinistrin have rarely been reported in the literature. These compounds have been implicated in rare allergic reactions to food (Gay-Crosier et al., 2000; Franck et al., 2005), and some patients presenting hypersensitivity or anaphylactic reaction to inulin or sinistrin after intravenous administration have been described (Chandra and Barron, 2002; Fux et al., 2004; Bacchetta et al., 2008a). The underlying mechanism of inulin or sinistrin hypersensitivity is not known.

In France, PROINULINE Serb® and INUTEST® are two sinistrin-based reagents used for glomerular filtration rate (GFR) measurement.

GFR is an important clinical indicator of kidney function. The GFR is the volume of fluid filtered out of the plasma through glomerular capillary walls into Bowman’s capsules per unit of time. The exact measurement of GFR requires an injection of an exogenous marker (i.e., Inuline) freely filtered at the glomerulus, and with no subsequent tubular modifications and the measurement of its plasma and urine concentrations.

INUTEST®, produced by Fresenius Kabi Austria GmbH, is imported by the SERB laboratory. Since May 2016, SERB has marketed PROINULINE Serb®, manufactured and controlled by Fresenius Kabi Austria GmbH using the same procedures employed for INUTEST®. Since 2016, the number of notifications of hypersensitivity reactions to sinistrin-based reagents has increased including serious cases of anaphylactic shock with life-threatening or fatal outcomes following the administration of PROINULINE Serb®. In 2018, the French National Agency for the Safety of Medicines and Health Products (*Agence Nationale de Sécurité du Médicament et des produits de santé*, ANSM) decided to withdraw all batches of PROINULINE Serb® and INUTEST®.

This study assessed the safety of inulin and sinistrin as active substances and diagnostic reagents. Multiple French sources were analyzed to identify and analyze adverse reactions (ARs) to inulin or sinistrin.

## Methods

### Study Design and Data Sources

This observational, retrospective, pharmacovigilance study is based on all cases of adverse reactions (ARs) to inulin and sinistrin from 1) classical pharmacovigilance databases with spontaneous reports of ARs, the French Pharmacovigilance database (FPVD) and the World Health Organization (WHO) Database VigiBase, 2) the MultiGFR clinical trial (Validation of New Markers of Glomerular Filtration Rate: Dota Gadolinium (Gd) and Calcium EDTA), a French national clinical trial and 3) patient records from a hospital.

Created in 1978, VigiBase is the World Health Organization (WHO) international database of suspected adverse drug reactions. It is the largest drug safety data repository in the world with over 25 million reports of suspected ARs of drugs. In France, since 1985, cases transmitted to the French Pharmacovigilance network are registered in the FPVD, that is administered by the ANSM. ARs reported by health professionals or patients are evaluated and validated by pharmacologists before registration in the FPDV.

Each report of AR includes the available data concerning the reporting country for Vigibase, the reporter’s type (physician, pharmacist, patient … ) the patient’s demographic characteristics, the drug(s) used and the characteristics of the adverse drug reactions. These reports originate from healthcare professionals, patients, or pharmaceutical companies, and are generally notified post-marketing.

### These Databases Contains Fully Anonymized Reports

Ethical review and approval was not required for this study on human participants in accordance with the local legislation and institutional requirements. We analyzed retrospectively anonymous pharmacovigilance cases from various databases.

### Data Extraction

We identified all cases related to inulin%, sinistrin%, and polyfructosan% reported before August 31, 2018. Inulin and sinistrin are coded under different terms in the FPVD including INULINE, POLYFRUCTOSAN, SINISTRINE, PRODUIT NON TRANSCODE, SPECIALITE NON TRANSCODEE; so we searched in the FPVD using each of these terms. To obtain an exhaustive list of cases, we also queried the VigiBase database, which includes French pharmacovigilance case reports transmitted by the French authorities and pharmaceutical companies to the WHO. We used in the VigiBase the keywords inulin, polyfructosan, sinistrin (substance), proinuline, and Inutest (trade name), and French cases submitted before August 31, 2018 were retrieved.

We crossed data found in FPVD and VigiBase in order to identify cases reported to both sources and cases only reported to FPDV or only to VigiBase. We merged these results to build a new database. The FPVD identification number was used to obtain detailed information about the patient (age, gender, past medical history), drug exposure, clinical description¸ AR characteristics (onset, seriousness), AR outcome. The causality was assessed through semiological and, chronological presentation.

We also collected information on all sinistrin-related ARs from the MultiGFR clinical trial. MultiGFR study is a clinical trial (ClinicalTrials.gov Identifier: NCT02286258) conducted to compare the performances of a reference tracer (sinistrin) and two novel non-radioactive tracers Calcium-EDTA and Gd-DOTA (Dotarem®) for GFR measurement in patients with chronic-kidney disease (CKD) and in healthy volunteers subjected to one or two GFR measurements. Participants ranged in age from 18 to 75 years and had no allergy history. We obtained a list of adverse event cases with detailed clinical information from clinical trial records.

Finally, we also had access to clinical data from the Department of Physiology of Necker Hospital, Paris, France, where routine GFR measurement by analysis of sinistrin renal clearance is performed. We collected medical records of patients who underwent a GFR measurement between January 2015 and December 2018 to assess all possible ARs that were not reported to the Pharmacovigilance network.

All potential ARs to inulin or sinistrin were analysed with special attention to hypersensitivity reactions and their correlations with specific batches of sinistrin. We took care to identify and eliminate duplicates between our sources before the final analysis, based on patient’s characteristics and ARs description.

## Definitions

Adverse event (AE): any untoward medical occurrence in a patient or clinical trial subject administered a medicinal product and which does not necessarily have a causal relationship with this treatment.

Adverse reaction (AR): a response to a medicinal product that is noxious and unintended. Response in this context means that a causal relationship between a medicinal product and an adverse event is at least a reasonable possibility. An adverse reaction, in contrast to an adverse event, is characterized by the fact that a causal relationship between a medicinal product and an occurrence is suspected.

An adverse reaction is considered serious if it meets one or more of the following criteria: results in death, or is life-threatening, requires inpatient hospitalization or causes prolongation of existing hospitalization, results in persistent or significant disability or incapacity, or, for exposure during pregnancy, results in a birth defect (HMA, 2011).

We classified the hypersensitivity cases based on their clinical description: immediate reactions are usually induced by an IgE-mediated mechanism and occur within the first hour following a drug administration. These reactions often involve (but not always) the skin or oral mucosa (such as lip or tongue swelling), the gastrointestinal system, the upper and lower respiratory tract, or cardiovascular system. The reaction can progress within minutes from the skin/oral mucosa to multiorgan involvement, abdominal cramps and vomiting, stridor, dyspnoea, wheezing, and circulatory collapse (due to massive volume loss into bodily tissues). Nonimmediate reactions may occur at any time from one to 48 h after the drug administration, and are often induced by a delayed T-cell dependent type of allergic reaction. Maculopapular exanthema is the most common non-immediate reaction (Lieberman, 2014).

### Statistical Analysis

All calculations were performed by using Microsoft® Excel®. Characteristics of cases were described in terms of mean or median [Min-Max] for quantitative variables, and in terms of effective and proportion for qualitative ones.

## Results

### Pharmacovigilance Databases

Within the two pharmacovigilance databases (FPVD and VigiBase) 134 cases related to inulin or sinistrin in France were reported from 1991 to 2018. The number of cases varied from 0 to 15 cases per year. There was an overrepresentation of cases from some centers, namely Lyon (66%), followed by Saint Etienne (10%), Paris (6%), Strasbourg (6%), Toulouse (5%), Lille (3%), and other cities (<1%). The median age of patients who had an AR was 40.5 years old [3–76]. Seventy-eight patients (58%) were male. Almost half of the cases were serious (47%). Hypersensitivity reactions were found in 129 patients (96%), the other five cases were digestive symptoms, including abnormal liver function tests. Hypersensitivity reactions to inulin or sinistrin varied from non-serious symptoms (skin or respiratory involvement) to anaphylactic shock, with 1–4 ARs described in the 129 patients (mean 1.6 (Cornelis, 2013; Sandilands et al., 2013; Mensink et al., 2015a; Shoaib et al., 2016)) as follow:

- One AR described per case (68 patients): anaphylactic shock (*n* = 6); acute hypotension or faintness (*n* = 10); bronchospasm or acute laryngeal symptom (*n* = 25); urticaria or facial edema (*n* = 27)
- Two ARs described per case (44 patients) mainly skin associated respiratory symptoms (20 cases) or urticaria associated with facial edema (6 cases), or urticaria with hypotension (2 cases), the overall cases were two skin and two respiratory symptoms (8 cases each)
- Three ARs described per case (13 patients), skin, respiratory and digestive symptoms (3 cases), urticarial, facial edema and respiratory symptoms (10 cases)
- Finally, 4 ARs for four patients: acute erythema with facial edema, dyspnea and faintness, close to anaphylactic shock.

The outcome was rapidly favorable in all cases, but one: a patient died of anaphylactic shock in 2018.

Inulin or sinistrin were the only suspected causes in 119 cases/134 (89% of all cases) and in 116 cases/129 (90% of hypersensitivity cases), and in all cases its causality assessment was probable (Table 1).

**TABLE 1:** Characteristics of 134 ARs to inulin and sinistrin registered in the FPVD and in the VigiBase from 1991 to 2018.

	No. of patients (% of the total) or median [Min-Max]
Total	134
Male/Female	78/56 (58 vs 42%)
Median age [Min-Max] (years)	40.5 [3–76]
Seriousness	63 (47%)
Hypersensitivity ARs	59 (44%)
Non-hypersensitivity ARs	4 (3%)
Hypersensitivity ARs	129 (96.3%)
Anaphylactic shock or related reaction	20 (15%)
Respiratory symptoms	25 (18.7%)
Skin symptoms	27 (20.1%)
Association of several symptoms	57 (42.5%)
Non-hypersensitivity ARs	5 (3.7%)
Digestive symptoms	3 (2.2%)
Liver	2 (1.5%)
Other drugs suspected	15 cases (11.2%)
Hypersensitivity ARs	13 cases (9.6%)
Other diagnosis agents, aminohippurique acid or iopromide (8 cases), Mannitol (5 cases)	
Non-hypersensitivity ARs	2 cases (0.6%)
Other the counter drugs for 2 cases of liver ARs	
Causality assessement	129 (96.3%)
Probable for all drugs for all hypersensitivity cases, with time to onset of less than a few minutes and suggestive clinical presentation	
Doubtful for other cases, poorly documented	5 (3.7%)

Results are presented as number (% of the total) or median [Min-Max] (Min: minimum; Max: maximum).

**FIGURE 1:**
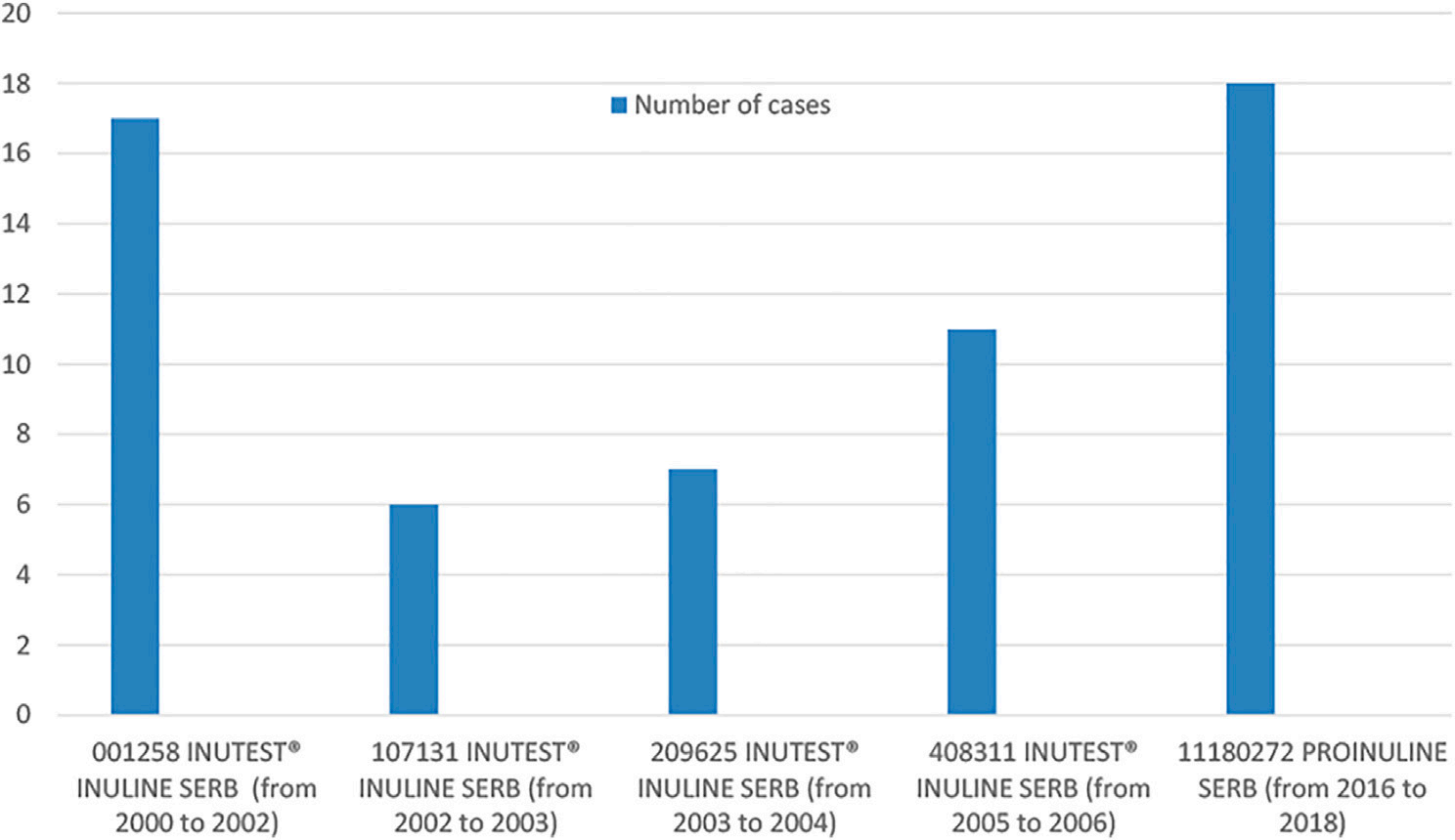
Distribution of cases in batches associated to the occurrence of 5 cases or more of ARs, based on batch number and year of occurrence.

Among the 96 cases with available information about batch number (72% of all cases and 73% of hypersensitivity cases), there were 29 different batches in total. Among these 29 batches, analyzing the number of cases reported to a specific batch, we found that 5 batches were associated to the occurrence of 5 cases or more of ARs and that two specific products and batches, PROINULINE Serb® number 11180272 and INUTEST® number 001258 had most ARs, all were hypersensitivity reactions (18 cases and 17 cases reported, respectively (Figure 1).

Regarding the years or period of cases occurrence, the higher number of cases (*n* = 24), were reported from 2016 to 2018. Among them, 21 cases (87.5%) concerned PROINULINE Serb®. Most of these cases (18/21), including the fatal case in Toulouse, received PROINULINE Serb®, batch number 11180272. This reagent was withdrawn from the French market in 2018.

## The MultiGFR Clinical Trial

The participants in the MultiGFR trial received INUTEST® from December 2014 to December 2016 and PROINULINE Serb® from January 2017. Among 163 participants (134 patients and 29 healthy volunteers), corresponding to 225 GFR measurements (62 participants were analyzed twice for reproducibility assessment), 19 participants (11.6%) experienced an AE. The number of AE reports was 21, because two participants had AEs during two different measurements.

After analysis of these 21 AE and evaluation of drug’s causality, we concluded that only seven reports (7/21 33%) were AR, and other AEs (67%) were related to causes such as a change of shaving foam or intercurrent disease. Among the 7 ARs related to inuline, four (57%) were hypersensitivity reactions, with one serious reaction that required immediate dedicated management: a grade 4 anaphylactic shock that occurred immediately after the injection of PROINULINE Serb® and Dotarem® with a favorable outcome following cardiopulmonary resuscitation. This case of grade 4 anaphylactic shock had one year later an allergic assessment that confirmed the role of PROINULINE Serb®. These allergic tests were carried out with the same batch of PROINULINE Serb® as the one administered to the patient that resulted in the shock reaction, and the skin test to Dotarem® was negative. Importantly, the batch of PROINULINE Serb® (number 11180272) that caused this serious AR was the same batch given to the patient in Toulouse that resulted in death and given to the other 17 AR cases reported to FPDV from 2016 to 2018 in Lyon, Saint Etienne, Toulouse, and Strasbourg. Of note, the patient who presented the grade 4 anaphylactic shock was the first and the only one patient included in the MultiGFR trial who received this batch of PROINULINE Serb® because the MultiGFR study was immediately and definitively suspended due to this serious AR. The other three cases were headaches with positive rechallenge in the same patient who had two measurement and one case of faintness (Table 2).

**TABLE 2:** Characteristics of ARs in the MultiGFR clinical trial.

	No. of patients (% of the total) or median [Min-Max]
Total	163
Male/Female	115/48
Median age [Min-Max] (years)	50 [21–76]
Number of AE	21
Number of AR	7
Seriousness	1
Hypersensitivity ARs	4
Non-hypersensitivity ARs	3
Hypersensitivity ARs	4
Anaphylactic shock or related reaction	1
Skin symptoms	2
Association of several symptoms	1
Non-hypersensitivity ARs	**3**
Headache	2
Faintness	1
Other drugs suspected	non applicable
Causality assessement	Probable for inuline for all ARs

Results are presented as number (% of the total) or median [Min-Max] (Min: minimum; Max: maximum).

### The Physiology Department at Necker Hospital

In the Physiology Department of the Necker Hospital, 57 patients had GFR measured using sinistrin (INUTEST®) clearance between January 2015 and December 2018. The number of measurements was 72 because some patients had GFR measured several times (twice, *n* = 13; 3 times, *n* = 1). Among these 57 patients, 37 patients (65%) were women. The median age was 51 years old [15–80], and 40 patients (70%) had had a kidney transplant. None of the 57 patients presented or reported an AR. Over the three-year period, the INUTEST® batches used were numbers 10851125, 10981190, 11089320, and 11180272.

## Discussion

We conducted a systematic analysis of all sources available (i.e., pharmacovigilance databases, a clinical trial, and real-world data) to assess the safety of inulin and sinistrin. To our knowledge, this is the first time that such an evaluation has been performed. In this retrospective analysis of 28 years of inulin and sinistrin use in France, the annual number of AR registered in the pharmacovigilance system varied from 0 to 15 cases with a reporting rate of ARs range from 0 to 2.54 cases per 10 million inhabitants. This reporting rate was low and may underestimate the true rate due to the expected underreporting of ARs. The low reporting rate may also be a reflection of the limited use of inulin and sinistrin: Only a few centers in France use these products for the measurement of GFR. Indeed, the ARs associated with inulin or sinistrin were reported to 5 of the 31 regional pharmacovigilance centers. This geographical bias reflects the distribution of the measurement of GFR by inulin or sinistrin renal clearance in France, which were not used everywhere. The overrepresentation of cases in one center (Lyon) is explained by a specific study of the tolerance of inulin that was reported in 2008 (Bacchetta et al., 2008b).

Although inulin is widely used in the food industry, only a few cases of hypersensitivity to inulin have been reported in the literature. Bacchetta et al. (2008b) systematically collected all cases of hypersensitivity reactions during GFR measurement of inulin clearance reported from January 1, 1998 to July 31, 2001 and from January 1, 2003 to December 31, 2006. For these two periods, 31 (0.49%) and 26 (0.43%) patients experienced AR, respectively. No life-threatening complication was observed.

Our analysis showed that a single batch of PROINULINE Serb® (number 11180272) was associated with 75% of AR events (18/24 cases) reported from 2016 to 2018 including the fatal case in Toulouse and the grade 4 anaphylaxis case from the MultiGFR clinical trial. These are the most serious ARs observed in France over a period of more than 20 years with this reagent. In contrast, no serious ARs were identified by Bacchetta et al. (Bacchetta et al., 2008b).

This raises questions about the quality of the medicinal product. Sinistrin is manufactured in large quantities from red squill. Chicory root is the main source of extraction for production process of the commercial inulin. The isolation process consists of three steps (extraction, purification, and finally spray drying). The quality of a product extracted from plants depends on the purification process to remove impurities. Pectic polysaccharides and inorganic non-sugars must be removed during purification as their presence directly affects the quality of the final product (Spies et al., 1992; Mensink et al., 2015b). Thus, an impurity remaining in certain batches might be the cause of ARs. Analysis of the manufacturing processes and possible recent changes, the quality control accuracy should be explored, regarding these recent and serious AR occurrence with some batches.

Of cases we identified, 47% were serious, including one fatal outcome and a serious anaphylactic shock with a favorable outcome following cardiopulmonary resuscitation. We thus did not come to the same conclusion as Bacchetta et al. who found that “hypersensitivity to inulin is not serious” (Bacchetta et al., 2008b). These apparently contradictory results highlight the determining role of production in the occurrence of AR to drugs and diagnostic reagents.

A limitation of this study was the use of pharmacovigilance databases. These resources can be biased due to underreporting of ARs. Further, certain cases were poorly documented in the FPVD. A strength is that in comparison to other studies, we analyzed a larger number of AR cases. Whereas most previous studies of the tolerance of inulin and sinistrin were case reports, our study is the largest study on this subject so far and is based on different sources, not only on spontaneous reporting in pharmacovigilance databases but also data from both clinical trial and observational cohorts, and thus more accurately assessed the hypersensitivity to inulin and sinistrin.

## Conclusion

In our study, we found that the majority of ARs associated with Insulin and Sinistrin used as diagnostic reagents for measurement of GFR were hypersensitivity reactions. If the Pharmacovigilance reports are not appropriate to evaluate the true reporting rate of these ARs, the reporting rate of hypersensitivity ARs during the clinical trial was 2.5%. We found that a few batches of sinistrin were associated with several hypersensitivity reactions, including the most serious. This issue questions the quality and purifications pf the product, extracted from plants.

## Data Availability

The raw data supporting the conclusion of this article will be made available by the authors, without undue reservation.

## References

[B1] Bacchetta J. Cochat P. Villard F. Astier M. Vial T. Dubourg L. (2008). Hypersensitivity to Inulin: a Rare and Mostly Benign Event. Am. J. Kidney Dis. 52 (3), 632–633. Sep. 10.1053/j.ajkd.2008.06.008 18725028

[B2] Bacchetta J. Villard F. Vial T. Dubourg L. Bouvier R. Kassaï B. (2008). ‘Renal Hypersensitivity' to Inulin and IgA Nephropathy. Pediatr. Nephrol. 23 (10), 1883–1885. Oct. 10.1007/s00467-008-0819-9 18535847

[B3] Chandra R. Barron J. L. (2002). Anaphylactic Reaction to Intravenous Sinistrin (Inutest). Ann. Clin. Biochem. 39 (Pt 1), 76. Jan. 10.1258/0004563021901621 11853197

[B4] Cornelis P. (2013). « Guide de Bonnes Pratiques en Culture de Chicorée Industrielle ». Belgium: Centre Agricole Betteraves Chicorées. Available at: https://www.irbab-kbivb.be/wp-content/uploads/2015/10/guide_bonnes_pratiques.pdf .

[B5] Franck P. Moneret-Vautrin D. A. Morisset M. Kanny G. Mégret-Gabeaux M. L. Olivier J. L. (2005). Anaphylactic Reaction to Inulin: First Identification of Specific IgEs to an Inulin Protein Compound. Int. Arch. Allergy Immunol. 136 (2), 155–158. Feb. 10.1159/000083323 15650313

[B6] Fux R. Biedermann T. Sander-Wiecker T. Mörike K. Gleiter C. H. (2004). Anaphylaxis to Intravenous Sinistrin. Ann. Pharmacother. 38 (12), 2175–2176. Dec. 10.1345/aph.1E267 15536136

[B7] Gay-Crosier F. Schreiber G. Hauser C. (2000). Anaphylaxis from Inulin in Vegetables and Processed Food. N. Engl. J. Med. 342 (18), 1372. May 4. 10.1056/NEJM200005043421814 10798950

[B8] HMA (2011). Guideline on Good Pharmacovigilance Practices (GVP) – Annex I (Rev 4). EMA/876333/2011.

[B9] Lieberman P. L. (2014). Recognition and First-Line Treatment of Anaphylaxis. Epub 2013 Oct 1. PMID: 24384138. Am. J. Med. 127 (1 Suppl. l), S6–S11. Jan. 10.1016/j.amjmed.2013.09.008 24384138

[B10] Mensink M. A. Frijlink H. W. van der Voort Maarschalk K. Hinrichs W. L. (2015). Inulin, a Flexible Oligosaccharide I: Review of its Physicochemical Characteristics. Carbohydr. Polym. 130, 405–419. Oct 5. 10.1016/j.carbpol.2015.05.026 26076642

[B11] Mensink M. A. Frijlink H. W. van der Voort Maarschalk K. Hinrichs W. L. (2015). Inulin, a Flexible Oligosaccharide. II: Review of its Pharmaceutical Applications. Carbohydr. Polym. 134, 418–428. Dec 10. 10.1016/j.carbpol.2015.08.0224 26428143

[B12] Oettl K. Payerl D. Zitta S. Müller T. Estelberger W. Reibnegger G. (2004). Quantitative Analysis of Sinistrin in Serum with High-Performance Liquid Chromatography for Renal Function Testing. Anal. Biochem. 331 (1), 183–188. Aug 1. 10.1016/j.ab.2004.03.019 15246012

[B13] Sandilands E. A. Dhaun N. Dear J. W. Webb D. J. (2013). Measurement of Renal Function in Patients with Chronic Kidney Disease. Br. J. Clin. Pharmacol. 76 (4), 504–515. Oct. 10.1111/bcp.12198 23802624 PMC3791974

[B14] Shoaib M. Shehzad A. Omar M. Rakha A. Raza H. Sharif H. R. (2016). Inulin: Properties, Health Benefits and Food Applications. Carbohydr. Polym. 147, 444–454. Aug 20. 10.1016/j.carbpol.2016.04.020 27178951

[B15] Spies T. Praznik W. Hofinger A. Altmann F. Nitsch E. Wutka R. (1992). The Structure of the Fructan Sinistrin from Urginea Maritima. Carbohydr. Res. 235, 221–230. Nov 4. 10.1016/0008-6215(92)80090-N 1473105

